# Availability and Nutritional Composition of Street Food in Urban Central Asia: Findings From Almaty, Kazakhstan

**DOI:** 10.3389/ijph.2022.1604558

**Published:** 2022-04-25

**Authors:** Gabriela Albuquerque, Inês Lança de Morais, Marcello Gelormini, Sofia Sousa, Susana Casal, Olívia Pinho, Albertino Damasceno, Pedro Moreira, João Breda, Nuno Lunet, Patrícia Padrão

**Affiliations:** ^1^ EPIUnit—Instituto de Saúde Pública, Porto, Portugal; ^2^ Laboratório para a Investigação Integrativa e Translacional em Saúde Populacional (ITR), Porto, Portugal; ^3^ Nutrition, Physical Activity and Obesity Programme, Division of Noncommunicable Diseases and Life-Course, World Health Organization Regional Office for Europe, Copenhagen, Denmark; ^4^ Faculdade de Ciências da Nutrição e Alimentação, Universidade do Porto, Porto, Portugal; ^5^ REQUIMTE, Laboratório de Bromatologia e Hidrologia, Faculdade de Farmácia, Universidade do Porto, Porto, Portugal; ^6^ Departamento de Ciências da Saúde Pública e Forenses e Educação Médica, Faculdade de Medicina, Universidade do Porto, Porto, Portugal; ^7^ Faculty of Medicine, Eduardo Mondlane University, Maputo, Mozambique; ^8^ Centro de Investigação em Atividade Física, Saúde e Lazer, Universidade do Porto, Porto, Portugal; ^9^ WHO European Office for the Prevention and Control of Noncommunicable Diseases, WHO Regional Office for Europe, Moscow, Russia

**Keywords:** public health, central asia, Kazakhstan, nutritional value, ready-prepared foods, street food

## Abstract

**Objective:** To describe the availability and nutritional composition of commonly available street foods in Almaty, Kazakhstan.

**Methods:** 384 street food vending sites (in 10 public markets) were assessed; information on vending sites’ characteristics and food availability and samples of the most commonly available street foods (81 homemade; 40 industrial) were collected for chemical analysis.

**Results:** Fruit, beverages and food other than fruit were available in 1.0%, 47.4% and 92.7% of all vending sites. Homemade food other than fruit (e.g., bread, main dishes, snacks, pastries, sandwiches, and cakes) were available in 63.4% of stationary vending sites, while industrial (e.g., snacks, chocolate, cakes, and cookies) in 45.6% of them. Industrial foods were the most energy-dense [median kcal/100 g: 438.8 vs. 267.2, *p* < 0.001 (homemade)]. Traditional homemade dishes were high in sodium, reaching 2,248 mg/serving (*lagman*) and major contributors of protein and fat to energy content (*shashlik*: 22.8% from protein, 68.3% from fat). Industrial chocolate and homemade cake presented the highest saturated (14.6 g/serving) and *trans*-fat (3.20 g/serving) contents.

**Conclusion:** These findings advocate for the implementation of health promotion strategies targeted at vendors, consumers and other stakeholders.

## Introduction

Street food is widely available to several populations worldwide, especially in low and middle-income countries (LMIC) [1, 2]. It is part of the Central Asian food culture, reflecting locally available food products, practices and consumer preferences [3, 4], a legacy of the nomadic multicultural populations who travelled the ancient Silk Road, exchanging goods, namely food, between Asia and Europe [5].

Countries in this region are undergoing a transition from traditional to more globalized eating patterns. Throughout the last 4 decades, a decreased availability of cereal, roots and tubers and increased availability of animal products, sugar, sweeteners and vegetables has been observed [6]. In parallel, a high burden of noncommunicable diseases (NCDs) has been observed at the regional and national level [6, 7]. Kazakhstan has a high rate of premature mortality (648.31 per 100,000 population aged 30–69 years in 2012) [8]. The leading cause of death in the country is cardiovascular disease, accounting for 54% of all deaths, followed by cancer (15%) and other NCDs (10%) [9]. Unhealthy diet, excessive alcohol consumption, high systolic blood pressure and high body mass index have been identified as important risk factors for disease burden in Kazakhstan [10, 11].

Food consumption away from home is increasing in the region, fostered by socio-economic phenomena, such as urbanization and women empowerment [12]. Street food is a popular example at the global level, often replacing home meals. Its popularity has been attributed to low-cost, convenience, considerable portion sizes and taste. Ultra-processed foods, particularly snacks, constitute a considerable share of the street food products sold in the street [1, 2], which adds to the global public health concern on the rise of ultra-processed food [13, 14]. It has also been shown that street food is a significant contributor to excess intake of energy and nutrients in several urban settings worldwide [2]. However, there is a literature gap about the types of street food items available and their nutritional composition [2], especially in the region [15].

Furthermore, Kazakhstan is one of the countries in the World Health Organization (WHO) European region where a lack of representative diet-related surveys has been identified [8, 16]. Kazakhstan is a landlocked upper-middle-income country and the largest in Central Asia. The country has experienced positive economic development since the early 2000’s, with the annual GDP growth being 4.1% in 2017. In the same period, it was observed a sharp decrease in the proportion of the population living below the national poverty lines (from 46.7% in 2001 to 2.5% in 2017) and a positive growth of the urban population. In 2017, 54.8% of the population lived in urban areas [17]. The city of *Almaty*, the former capital, is the largest in the country, with an estimated population of approximately 1.8 million inhabitants, corresponding to approximately 10% of the country’s population [18]. This study aimed to characterize the street food environment in *Almaty*, Kazakhstan, focusing on the vending sites, food availability and the nutritional composition of the most commonly available street foods and beverages. It was implemented in the context of the FEEDcities project, supported by the WHO—Europe, which is based on a stepwise standardised methodology to describe the street food environment in countries in Central Asia and Eastern Europe [19].

## Methods

### Study Design

This cross-sectional evaluation was conducted between July and August 2017 in *Almaty*, Kazakhstan. The street food definition proposed by Food and Agriculture Organization and WHO was the one used in this study—“ready-to-eat foods and beverages prepared and/or sold by vendors or hawkers especially in the streets and other similar places” [20, 21]. From observations made by the research team before the study implementation, it was acknowledged that, in this city, most street food vendors were located within or in the proximity of public markets. Thus, from a list of 23 public markets identified by local authorities, 10 were randomly selected. The study area was delimited by a 500-m buffer around the centroid of each of the selected markets, covering them and their surroundings.

Eligible vending sites were defined as the establishments selling ready-to-eat food, including beverages and/or snacks, from any venue other than permanent storefront businesses or establishments with four permanent walls not selling directly to the street, operating in the predefined perimeter (including, thus, mobile, semi-static or stationary vending units). The exclusion criteria were: 1) food establishments with four permanent walls; 2) permanent storefront business; 3) street vendors selling non-food products or raw foods not ready-to-eat exclusively; 4) food stalls and carts that were part of permanent stores or licensed establishments.

### Data Collection

The markets were assessed on nine consecutive days, including weekdays and weekends. Pairs of field researchers canvassed each study area systematically to find street food vendors. The selection of the vending sites to be assessed was based on the estimated number of vending sites in each market: in those with 100 or less vending sites (*n* = 8), the vendors from all eligible vending sites were invited to participate, and in markets with more than 100 vending sites (*n* = 2), all the vending sites were mapped, but one in every two was systematically assessed, and the respective vendors were invited to participate [19].

The interviewers registered the Global Positioning System (GPS) coordinates and collected the following information, through direct observation at each vending site: sex of the vendor, mobility of the vending site and type of physical setup. Stationary vending sites were categorised into formal [stand, *dukoni* (pavement side window from a permanent storefront business or establishment with four permanent walls, traditional in Central Asia), table with chairs for customers or truck] or informal (bench with table, pushcart, and other improvised sites such as freezer machine, selling soft ice-cream or beverages such as lemonade).

Afterwards, the field researchers approached the vendor to explain the study objectives and procedures and ask for express consent to participate. Following the vendor’s agreement, they carried out computer-assisted personal interviewing, enquiring about vending site ownership, access to drinking water/toilet facilities and food availability, including serving sizes. Additional questions concerning vending sites’ characteristics (access to electricity) and food vending activity (operating periods; e.g., during the week, during the year and under which weather conditions) were asked only to vendors operating stationary vending sites, due to the limited time of mobile vendors to answer the questionnaire [19].

Of 816 eligible street food vendors identified, and 396 approached, 384 agreed to participate (97.0% participation). Stationary vendors were more likely to participate than mobile ones (92.7% vs. 75.0%, *p* = 0.006), but there were no other statistically significant differences between participant and non-participant vendors, by market or physical setups.

Foods available were classified into fruit (product in *natura*, either fresh or dry), beverages (any alcoholic and non-alcoholic drink) or food other than fruit, according to their nature. Food other than fruit was further grouped as homemade (foods of domestic manufacture cooked and/or prepared at home or on the street, even if using industrial ingredients) or industrial (foods produced by the food industry and sold as is without further preparation and/or cooking). Homemade food was also grouped according to the preparation method in cooked or uncooked, and also according to broader food groups, that were created based on the groups of the WHO nutrient profile model [22]: 1) bread; 2) cakes, biscuits and pastries; 3) main dishes and sandwiches; 4) savoury pastries, 5) snacks and 6) ice-cream, chocolate and confectionery. Beverages were categorised into soft drinks, water, fruit juice-based drinks, fresh fruit juice-based drinks, milk, energy drinks, coffee, tea, fruit smoothies (ice and natural fruit extract-based beverages) and traditional beverages (including *ayran*, *kephyr*, *yoghurt*, *kompot* and *kozhe*, non-alcoholic, and *kymys*, *shubat* and *kvas*, low alcoholic drinks).

### Food Sample Collection and Nutritional Composition Assessment

Subsequently to data collection, the frequency of all foods and beverages identified across the study sample was computed, and samples of the most commonly available street foods and beverages were collected for nutritional composition assessment. The most frequent homemade foods (*n* = 19) included bread (namely *lepyoshka* and *baursak*), traditional dishes (prepared salads such as cabbage salad, or meat-based dishes such as *plov*, *lagman* and *shashlik* and a meat-based traditional sandwich, *shawarma*), savoury pastries (baked or fried; filled with meat, vegetables or potato, such as sausage roll, *samsa*, *pirozhki*, *belyashi* and *chebureki*), snacks (such as boiled corn cob, *kurut—*a salty dairy-based snack made by straining and drying sour milk or yoghurt—and *jent*—a sweet snack made of a hard grain like millet, soaked in sugar and oil), cakes and pastries (bun, pancake and cake) and ice-cream. A traditional homemade non-alcoholic local beverage, *kozhe*, was also collected, given its wide availability in the city and unknown composition, adding to a total of 20 most frequent homemade street foods. The most frequent industrial foods (*n* = 10) included snacks (croutons, chips, cookies, dry bread rings, and popcorn), chocolate, wafers, cake, muffin (*keksi*), and gingerbread biscuit (*pryaniki*). Supplementary Table S2 provides brief descriptions and photographs of the traditional street foods collected.

The vending sites where the food samples collection was carried out were selected randomly, from the list of the GPS coordinates of the eligible vending sites previously identified (ten GPS coordinates—location of ten vending sites—randomly selected in each market). A sample of each food product, corresponding to one serving, was bought whenever possible at these vending sites. An alternative systematic selection procedure was followed when it was not possible, described in more detail in the FEEDcities protocol [19]. In each vending site, only one sample of homemade and one sample of industrial food or beverage could be obtained.

Four samples of all selected homemade and industrial foods and beverages were collected from different vending sites. Exceptionally, five samples of homemade cake were purchased. A total of 121 samples (81 homemade and 40 industrial) were collected in five consecutive days.

Samples were homogenized, weighted and stored in a freezer (−18°C) until the nutritional composition assessment [19], which included the analysis of: 1) moisture, by oven drying at 103°C until constant weight; 2) protein, by Kjedahl’s method, using a nitrogen conversion factor of 6.25; 3) fat, by Soxhlet’s method and 4) fatty acids [saturated (SFA), monounsaturated (MUFA), polyunsaturated (PUFA), n-3 and n-6 fatty acids, trans-fatty acids (TFA)], by gas chromatography for the fatty acid methyl esters [23]. Total carbohydrates plus fibre were estimated by difference. The samples’ energy value was estimated after proximate analysis of food components (moisture, protein, total fat, and ash), performed following standard recommendations [24]. Sodium and potassium were analysed by flame photometry [25]. The analytical results were the average of two determinations per food sample. A third determination was conducted if the coefficient of variation of the first two was above 5% for fatty acids and 1% for micronutrients. In this case, the average of two determinations consistent with these criteria was computed. All the analytical results were expressed by serving size (g).

### Statistical Analyses

The vending sites, vendors and food availability were described using absolute and relative frequencies (categorical variables) and compared using Pearson’s chi-square. Markets were defined as the sampling units. The statistical analyses were conducted adjusting for the clustering at the sampling unit level.

Concerning the presentation of the nutritional composition assessment results, mean serving sizes per food, in grams (g), were calculated as the mean weight of the individual samples collected for each of the foods. Per-serving levels of each nutrient were calculated as the mean of the individual samples and expressed in (g) or (mg)/serving (macronutrients or micronutrients, respectively). For each food, results were summarized as the mean and range of energy (kcal), macronutrients (g) and micronutrients (mg), as well as molar sodium-to-potassium (Na/K) ratios. Contents of sodium and potassium of each sample were converted into millimoles according to their molar weights (respectively, 23.0 and 39.1 g/mol), to calculate individual molar Na/K M ratios. The Mann–Whitney *U* test was used for comparing the energy content of homemade and industrial foods. The critical level of significance (p) was defined as less than 0.05. Statistical analyses were performed using STATA^®^, version 15.1 (StataCorp, College Station, TX, United States).

## Results

### Food Vending Sites and Vendors

**FIGURE 1:**
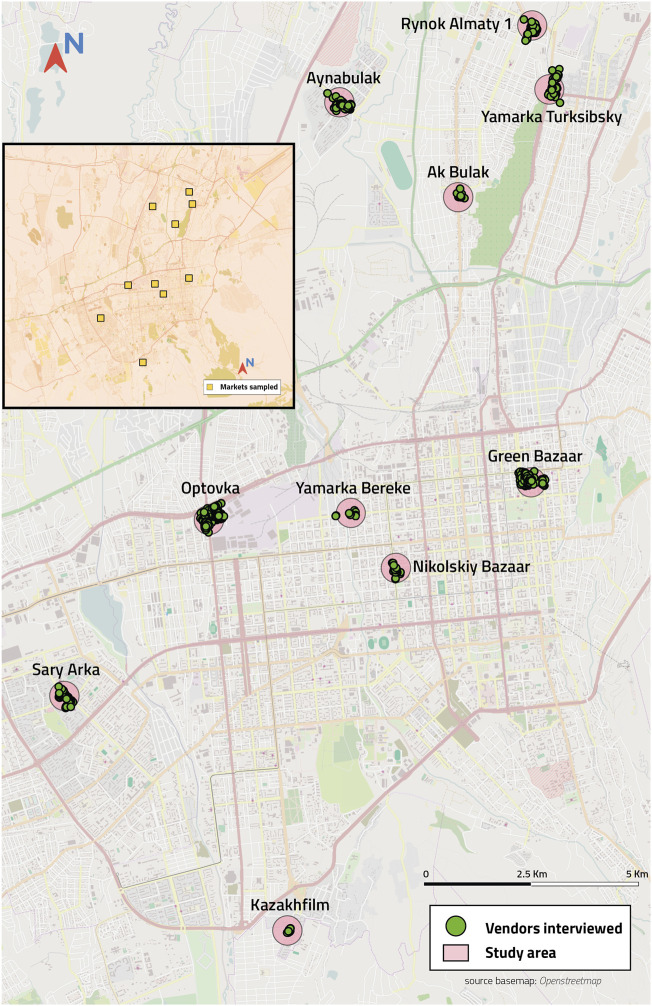
Geographical distribution of the markets and street food vending sites (Almaty, Kazakhstan. 2017).

Figure 1 depicts the distribution of the selected markets, and respective vending sites throughout the *Almaty* city.

Almost all vending sites were stationary (92.7%) (Supplementary Table S1), namely *dukoni* (59.6%) and stands (29.2%). All stationary vending sites had access to drinking water and most had access to toilet facilities (99.2%) and electricity (91.9%), were operating under every type of weather (97.2%) and 6 days a week (94.7%), all year long (91.0%). The informal ones were less likely to be operating the whole year (50.0% vs. 94.2%, *p* < 0.001) (Table 1). Approximately three in every four street food vendors were women (74.2%), and one third were owners of the business (32.8%), except in mobile vending sites, where most vendors (71.4%) were owners.

**TABLE 1:** Characteristics of street food vending sites and food availability, by type of stationary vending site (*n* = 356) (Almaty, Kazakhstan. 2017).

	Total (*n* = 356)		Physical Setup^d^				*p*
			Formal (*n* = 330; 92.7%)		Informal (*n* = 26; 7.3%)		
Vendors and vending sites characteristics							
Food vendor sex (women)	265	74.4	247	74.9	18	69.2	0.599
Vendor owns the vending site	106	29.8	96	29.1	10	38.5	0.671
Infrastructure							
Access to drinking water^a^	354	100.0	328	100.0	26	100.0	
Access to toilet facility	353	99.2	329	99.7	24	92.3	0.005^e^
Access to electricity	327	91.9	312	94.6	15	57.7	0.005^e^
Business operating time							
Operating 6 days/week	337	94.7	311	94.2	26	100.0	0.734
Operating the whole year	324	91.0	311	94.2	13	50.0	<0.001^e^
Operating under every type of weather	346	97.2	321	97.3	25	96.2	0.808
Food availability							
Fruit	4	1.1	4	1.2	0	0.0	0.704
Food other than fruit	333	93.5	312	94.6	21	80.8	0.014^e^
Industrial^a^	122	36.6	118	37.8	4	19.1	0.039^e^
Homemade and Industrial^a^	30	9.0	30	9.6	0	0.0	
Homemade^a^	181	54.4	164	52.6	17	81.0	
Preparation of homemade foods^b^							
Cooked	163	77.3	154	79.4	9	52.9	0.121
Uncooked	80	37.9	71	36.6	9	52.9	0.421
Beverages	166	46.6	159	48.2	7	26.9	0.026^e^
Soft drinks	102	61.5	99	62.3	3	42.9	0.385
Traditional beverages^c^	86	51.8	84	52.8	2	28.6	0.304
Water	80	37.9	71	36.6	9	52.9	0.771
Energy drinks	55	33.1	55	34.6	0	0.0	0.117
Tea	39	23.5	39	23.5	0	0.0	0.138
Coffee	32	19.3	32	20.1	0	0.0	0.223
Fruit juice-based drink	39	23.5	39	24.5	0	0.0	0.165
Milk	20	12.1	20	12.6	0	0.0	0.317
Fresh fruit juice-based drink	5	3.0	4	2.5	1	14.3	0.075
Fruit smoothies	13	7.8	12	7.6	1	14.3	0.249

a Sample size is lower for this variable due to missing data.

b The sum of the values for this variable is higher than the total number of homemade foods, as each vendor could offer foods prepared in different ways.

c Traditional beverages: non-alcoholic—*ayran* (*n* = 43), *kompot* (*n* = 24), *kozhe* (*n* = 10), *kephyr* (*n* = 10) and *yoghurt* (*n* = 10); low alcoholic content (<0.6%): *kymys* (*n* = 17), *shubat* (*n* = 17) and *kvas* (*n* = 12).

d Informal physical setups: freezer machine (*n* = 12)**;** bench with table board (*n* = 9); pushcart (*n* = 5). Formal physical setups: dukoni (*n* = 212); stand (*n* = 104); truck (*n* = 13) and tables with chairs for customers (*n* = 1).

e Statistically significant differences according to Pearson’s Chi-square test, for a confidence level of 95% (*p*-value<0.05).

### Food Availability

Fruit was available in 1.0%, while beverages and food other than fruit were available, respectively, in 47.4% and 92.7% of the vending sites (Supplementary Table S1). Among stationary vending sites, 54.4% sold only homemade, 36.6% only industrial and 9.0% both homemade and industrial foods. Most homemade foods other than fruit were cooked (77.3%). Main dishes and sandwiches, savoury pastries and bread were the most commonly available groups of homemade foods, in at least 30% of these vending sites. Ice-cream, chocolate and confectionery, snacks and bun, cakes, cookies and sweet pastries were the most frequent groups of industrial foods, available in over 60% of the stationary vending sites selling industrial foods. Soft drinks (61.5%), traditional beverages (51.8%) and water (37.9%) were the top three most available beverages (Table 1).

### Nutritional Composition

Overall, homemade foods were less energy-dense than industrial foods (median kcal/100 g: 267 vs. 439, *p* < 0.001), but no significant differences were found in the energy content per serving. By food group, excluding the only collected beverage, the mean energy content per serving was highest in traditional homemade dishes (482 kcal/serving), ranging from 94 kcal/serving in cabbage salad*,* to 845 kcal/serving in *shawarma*, and lowest in homemade snacks (166 kcal/serving), ranging from 98 kcal/serving in *kurut* to 234 kcal/serving in boiled corn cob.

Traditional meat-based homemade dishes presented the highest mean contents in protein (lagman: 29.7 g/serving, *shawarma*: 33.9 g/serving), carbohydrates (*lagman*: 109.9 g/serving, *plov*: 117.9 g/serving) and total fat (*plov:* 28.6 g/serving, *shawarma*: 35.6 g/serving). The lowest mean contents in protein (cabbage salad: 1.1 g/serving) and carbohydrates (*shashlik*: 6.9 g/serving) were also found in the group of homemade dishes. *Kozhe*, a traditional non-alcoholic homemade beverage, was the product with the lowest content in total fat (0.3 g/serving) (Table 2).

**TABLE 2:** Nutritional composition (energy and macronutrients) of the street food samples collected, per serving (Almaty, Kazakhstan. 2017).

	N	Mean Serving Size (min–Max) (g/serving)		Mean Energy (min–Max) (kcal/serving)		Mean Protein (min–Max) (g/serving)		Mean Carbohydrates (min–Max) (g/serving)		Mean total Fat (min–Max) (g/serving)		Mean Water (min–Max) (g/serving)	
Industrial Food													
Biscuits	4	38	(30–46)	182	(144–208)	2.9	(2.1–3.5)	44.2	(36.8–47.4)	7.9	(5.9–11.6)	1.6	(0.9–2.8)
Cake	4	77	(60–92)	316	(277–374)	5.2	(3.9–8.8)	46.8	(37.5–57.8)	13.2	(7.9–18.7)	13.7	(2.4–27.5)
Chips	4	40	(40–40)	220	(215–224)	1.7	(1.6–1.9)	22.9	(22.3–23.7)	13.5	(12.5–14.2)	0.4	(0.3–0.6)
Chocolate	4	94	(89–100)	501	(455–551)	6.1	(4.8–7.5)	59.1	(53.1–63.8)	26.7	(21.3–32.6)	0.9	(0.6–1.2)
Croutons	4	36	(28–48)	143	(114–185)	3.9	(2.9–6.0)	25.0	(19.5–31.8)	1.8	(0.8–2.3)	1.7	(1.0–2.4)
Dry bread rings	4	33	(22–36)	132	(88–147)	3.2	(2.2–3.7)	27.9	(21.3–38.4)	2.2	(1.6–2.5)	2.1	(1.6–2.6)
*Gingerbread cookie (Pryaniki)*	4	50	(40–60)	188	(151–223)	3.3	(2.4–4.2)	37.2	(31.8–44.3)	2.9	(1.6–4.1)	6.3	(4.1–8.8)
Muffin (*keksi*)	4	76	(58–99)	338	(227–422)	4.4	(2.4–5.5)	24.6	(16.2–27.7)	14.8	(6.3–18.7)	8.9	(3.0–15.8)
Popcorn	4	63	(63–63)	252	(250–257)	3.0	(2.3–4.1)	55.0	(53.9–56.5)	2.2	(1.6–2.8)	2.1	(1.6–2.8)
Wafers	4	54	(52–56)	279	(268–289)	4.7	(2.9–7.4)	33.7	(32.2–37.3)	13.9	(12.5–14.8)	0.9	(0.7–1.5)
Homemade food													
*Belyashi*	4	119	(95–137)	322	(203–405)	8.1	(7.1–9.3)	48.1	(41.3–53.6)	10.8	(1.0–17.1)	51.1	(44.3–55.6)
Bread (*Baursak*)	4	63	(49–74)	227	(175–273)	4.4	(3.3–5.0)	33.4	(23.8–41.5)	8.4	(5.1–11.4)	16.2	(13.6–20.8)
Bread (*Lepyoshka*)	4	60	(43–70)	186	(123–253)	5.5	(3.6–6.6)	35.1	(24.3–40.7)	2.7	(0.1–7.4)	16.1	(10.0–22.7)
Bun	4	96	(35–146)	326	(99–488)	7.0	(2.3–11.2)	56.0	(22.3–88.1)	8.2	(0.0–25.1)	24.1	(9.8–43.7)
Cake	5	118	(61–190)	471	(246–810)	5.3	(2.1–7.9)	72.7	(40.2–110.1)	17.6	(6.7–37.6)	21.0	(9.6–34.8)
*Chebureki*	4	124	(103–139)	412	(337–452)	9.6	(7.7–10.5)	51.8	(43.5–60.5)	18.4	(14.7–20.4)	41.6	(33.8–60.0)
Corn cob	4	220	(151–385)	234	(154–395)	6.7	(4.8–10.1)	42.8	(26.5–75.2)	4.0	(2.5–6.0)	165.2	(114.2–290.2)
Ice-cream	4	132	(93–166)	217	(122–289)	5.1	(2.5–6.7)	43.9	(26.5–57.7)	2.3	(0.7–3.6)	79.5	(62.9–96.5)
*Jent*	4	60	(60–60)	300	(293–312)	2.7	(2.2–3.0)	39.5	(38.0–41.4)	14.6	(13.4–16.5)	2.9	(1.9–4.0)
*Kozhe*	4	200	(200–200)	42	(25–63)	2.1	(1.7–2.8)	7.8	(3.3–12.9)	0.3	(0.1–0.5)	187.9	(182.0–192.5)
*Kurut*	4	31	(22–40)	98	(73–120)	8.0	(1.4–12.3)	9.4	(3.1–17.6)	3.1	(1.0–6.5)	7.6	(3.7–10.4)
*Lagman*	4	524	(438–635)	697	(623–786)	29.7	(20.2–39.8)	104.9	(88.1–115.8)	17.6	(7.2–22.5)	364.9	(279.8–472.3)
Pancakes	4	107	(77–136)	242	(194–319)	7.0	(4.0–11.7)	37.8	(32.3–52.7)	6.1	(4.4–7.7)	52.4	(35.0–62.9)
*Pirozhki*	4	123	(111–130)	346	(302–376)	6.9	(6.5–7.2)	49.7	(48.7–51.1)	13.3	(8.3–17.0)	51.4	(37.1–63.4)
*Plov*	4	406	(352–479)	821	(672–982)	23.1	(19.0–30.5)	117.9	(94.1–131.5)	28.6	(23.7–37.3)	229.2	(207.4–287.2)
Salad (cabbage)	4	90	(69–112)	94	(60–131)	1.1	(0.8–1.8)	8.3	(5.3–11.4)	6.3	(3.1–9.9)	72.7	(55.6–89.7)
*Samsa*	4	96	(79–112)	297	(228–357)	8.1	(6.9–10.9)	40.3	(38.7–44.5)	11.5	(2.2–17.5)	34.1	(16.4–43.1)
Sausage roll	4	99	(67–134)	262	(194–337)	9.3	(6.8–11.6)	41.4	(25.2–73.3)	6.5	(0.4–9.4)	40.3	(26.4–49.7)
*Shashlik*	4	96	(58–126)	314	(205–390)	17.9	(13.0–24.7)	6.9	(3.0–12.0)	23.9	(15.6–31.3)	44.8	(24.9–64.0)
*Shawarma/Doner* k*ebab*	4	385	(353–429)	845	(693–914)	33.9	(24.0–41.7)	97.3	(91.9–103.7)	35.6	(25.5–41.6)	210.8	(171.0–245.1)

Homemade meat-based traditional dishes (*shashlik:* 12.3 g SFA/serving; *shawarma:* 13.2 g MUFA/serving) and industrial chocolate (14.6 g SFA/serving and 10.5 g MUFA/serving) presented the highest mean contents in SFA and MUFA. The former also presented the highest content in PUFA, mainly n-6 fatty acids, reaching 14.6 g/serving in *plov*, a vegetable and meat-based homemade dish. The amount of n-3 fatty acids in the analysed foods was very low, not exceeding 0.4 g/serving (in industrial wafers and homemade *shashlik*). *Samsa*, a homemade fried savoury pastry, and homemade cake presented the highest TFA content (2.14 and 3.20 g/serving, respectively) (Table 3).

**TABLE 3:** Nutritional composition (fatty acid profile) of the street food samples collected, per serving (Almaty, Kazakhstan. 2017).

	N	Mean Serving Size (min–Max) (g/serving)		Mean SFA (min–Max) (g/serving)		Mean MUFA (min–Max) (g/serving)		Mean PUFA (min–Max) (g/serving)		Mean n-6 (min–Max) (g/serving)		Mean n-3 (min–Max) (g/serving)		Mean TFA (min–Max) (g/serving)	
Industrial Food															
Biscuits	4	38	(30-46)	3.1	(2.2–3.7)	2.7	(2.2–3.3)	1.9	(0.8–4.3)	1.8	(0.8–4.2)	0.0	(0.0–0.0)	0.24	(0.04–0.44)
Cake	4	77	(60-92)	5.2	(3.7–6.9)	3.6	(1.9–4.9)	3.0	(1.0–5.5)	2.9	(0.9–5.4)	0.1	(0.0–0.1)	1.36	(0.33–2.37)
Chips	4	40	(40-40)	3.9	(1.5–6.4)	4.9	(3.6–5.8)	4.6	(1.6–7.5)	4.5	(1.6–7.5)	0.0	(0.0–0.1)	0.16	(0.08–0.22)
Chocolate	4	94	(89-100)	14.6	(9.9–20.5)	10.5	(7.7–13.0)	1.4	(1.2–1.6)	1.3	(1.1–1.5)	0.1	(0.1–0.1)	0.17	(0.07–0.29)
Croutons	4	36	(28-48)	0.2	(0.2–0.2)	0.6	(0.2–1.3)	0.5	(0.3–0.7)	0.8	(0.3–1.3)	0.0	(0.0–0.2)	0.03	(0.01–0.07)
Dry bread rings	4	33	(22–36)	0.8	(0.5–1.0)	0.7	(0.5–0.8)	0.9	(0.3–1.3)	0.4	(0.3–0.7)	0.0	(0.0–0.0)	0.29	(0.18–0.47)
*Gingerbread cookie (pryaniki)*	4	50	(40–60)	0.6	(0.3–0.9)	0.8	(0.5–1.1)	1.0	(0.5–2.4)	1.0	(0.5–2.4)	0.0	(0.0–0.0)	0.36	(0.02–0.71)
Muffin (*keksi*)	4	76	(58–99)	5.9	(0.7–10.3)	5.0	(2.9–6.6)	2.6	(1.4–3.6)	2.5	(1.4–3.5)	0.1	(0.0–0.1)	1.37	(0.01–3.99)
Popcorn	4	63	(63–63)	1.1	(0.3–1.4)	0.4	(0.1–0.7)	0.7	(0.1–1.6)	0.7	(0.1–1.6)	0.0	(0.0–0.0)	0.04	(0.00–0.13)
Wafers	4	54	(52–56)	5.1	(2.6–6.8)	5.3	(3.3–7.3)	1.5	(0.2–3.1)	1.1	(0.2–1.6)	0.4	(0.0–1.5)	1.98	(1.03–3.52)
Homemade food															
*Belyashi*	4	119	(95–137)	1.7	(0.2–2.5)	2.9	(0.3–4.4)	5.9	(0.5–10.3)	5.9	(0.5–10.3)	0.0	(0.0–0.1)	0.24	(0.01–0.57)
Bread (*Baursak*)	4	63	(49–74)	1.1	(0.7–1.8)	2.0	(1.0–2.8)	5.1	(3.0–7.1)	5.1	(3.0–7.1)	0.0	(0.0–0.0)	0.18	(0.12–0.28)
Bread (*Lepyoshka*)	4	60	(43–70)	0.4	(0.0–0.8)	0.7	(0.0–2.0)	1.3	(0.1–4.5)	1.3	(0.1–4.4)	0.0	(0.0–0.0)	0.20	(0.00–0.73)
Bun	4	96	(35–146)	3.4	(0.0–11.5)	2.5	(0.0–8.0)	1.9	(0.0–4.2)	1.9	(0.0–4.1)	0.0	(0.0–0.1)	0.47	(0.00–1.51)
Cake	5	118	(61–190)	4.7	(1.0–12.1)	5.6	(2.1–11.6)	4.2	(1.4–9.4)	4.0	(1.3–9.2)	0.1	(0.0–0.3)	3.20	(0.06–9.18)
*Chebureki*	4	124	(103–139)	3.2	(2.2–4.8)	5.3	(4.3–6.1)	9.6	(7.1–11.5)	9.5	(7.1–11.4)	0.1	(0.0–0.1)	0.36	(0.15–0.57)
Corn cob	4	220	(151–385)	0.9	(0.5–1.4)	1.2	(0.2–1.9)	2.1	(1.3–2.8)	1.4	(0.0–2.6)	0.1	(0.0–0.1)	0.22	(0.00–0.84)
Ice-cream	4	132	(93–166)	1.4	(0.4–2.3)	0.6	(0.2–0.9)	0.2	(0.1–0.3)	0.1	(0.0–0.2)	0.0	(0.0–0.1)	0.12	(0.03–0.18)
*Jent*	4	60	(60–60)	6.0	(4.9–7.7)	4.3	(3.4–5.3)	3.2	(3.1–3.5)	3.1	(2.9–3.2)	0.1	(0.0–0.2)	1.11	(0.35–1.40)
*Kozhe*	4	200	(200–200)	0.1	(0.0–0.3)	0.1	(0.0–0.2)	0.0	(0.0–0.1)	0.0	(0.0–0.1)	0.0	(0.0–0.0)	0.01	(0.00–0.03)
*Kurut*	4	31	(22–40)	1.7	(0.5–3.0)	0.9	(0.3–2.0)	0.2	(0.1–0.7)	0.2	(0.0–0.6)	0.0	(0.0–0.1)	0.30	(0.04–0.81)
*Lagman*	4	524	(438–635)	3.8	(1.7–5.9)	4.8	(3.0–6.0)	8.6	(2.5–14.3)	5.0	(2.3–14.1)	0.1	(0.1–0.2)	0.35	(0.08–0.56)
Pancakes	4	107	(77–136)	1.2	(0.7–2.2)	1.7	(1.2–2.3)	3.0	(2.3–3.7)	3.0	(2.3–3.6)	0.0	(0.0–0.1)	0.10	(0.03–0.21)
*Pirozhki*	4	123	(111–130)	1.7	(1.2–2.2)	3.1	(1.6–5.5)	8.3	(5.3–11.0)	8.2	(5.3–10.9)	0.0	(0.0–0.1)	0.19	(0.12–0.28)
*Plov*	4	406	(352–479)	5.5	(3.4–7.5)	7.8	(5.6–11.7)	14.6	(12.6–17.3)	14.5	(12.5–17.1)	0.1	(0.1–0.2)	0.59	(0.42–0.72)
Salad (cabbage)	4	90	(69–112)	0.7	(0.4–1.2)	1.4	(0.7–2.4)	4.1	(1.9–6.7)	4.0	(1.9–6.7)	0.0	(0.0–0.0)	0.07	(0.03–0.15)
*Samsa*	4	96	(79–112)	4.5	(0.7–7.8)	3.3	(0.6–5.8)	1.6	(0.8–3.5)	1.4	(0.7–3.4)	0.1	(0.0–0.2)	2.14	(0.04–4.80)
Sausage roll	4	99	(67–134)	1.6	(0.1–2.2)	2.2	(0.1–3.1)	2.6	(0.2–4.1)	2.5	(0.2–4.0)	0.1	(0.0–0.2)	0.16	(0.01–0.38)
*Shashlik*	4	96	(58–126)	12.3	(7.9–16.6)	8.5	(5.7–10.9)	1.2	(1.0–1.5)	0.7	(0.5–1.0)	0.4	(0.3–0.5)	1.87	(1.03–2.61)
*Shawarma/Doner kebab*	4	385	(353–429)	7.8	(5.4–9.5)	13.2	(9.6–16.3)	14.2	(10.2–16.4)	13.8	(10.0–16.1)	0.2	(0.2–0.3)	0.45	(0.18–0.72)

SFA, saturated fatty-acids; MUFA, monounsaturated fatty-acids; PUFA, polyunsaturated fatty-acids; TFA, trans-fatty acids

Mean sodium content ranged between 11 mg/serving in homemade *jent* and 2,248 mg/serving in homemade *lagman*, whereas mean potassium content ranged between 44 mg/serving in industrial biscuits and 1,327 mg/serving in homemade *shawarma*. A high mean sodium/potassium molar ratio was found in most homemade and industrial foods, ranging from 0.3 in homemade *jent* to 25.1 in homemade *kurut* (Table 4).

**TABLE 4:** Nutritional composition (sodium, potassium and molar sodium-potassium ratio) of the street food samples collected, per serving (Almaty, Kazakhstan. 2017).

Industrial Food	N	Mean Serving Size (min–Max) (g/serving)		Mean Na (min–Max) (mg/serving)		Mean K (min–Max) (mg/serving)		Mean Na:K (min–Max)	
Cookies	4	38	(30–46)	98	(1–138)	44	(30–55)	3.5	(0.0–5.0)
Cake	4	77	(60–92)	90	(27–129)	101	(78–145)	1.5	(0.5–2.8)
Chips	4	40	(40–40)	239	(82–348)	194	(86–243)	2.6	(0.7–5.3)
Chocolate	4	94	(89–100)	99	(45–213)	415	(239–671)	0.6	(0.2–1.5)
Croutons	4	36	(28–48)	262	(109–453)	111	(59–143)	3.8	(2.3–5.5)
Dry bread rings	4	31	(22–36)	119	(72–178)	62	(58–67)	3.3	(2.0–5.2)
Gingerbread cookie (*pryaniki*)	4	50	(40–60)	57	(7–111)	87	(37–144)	1.1	(0.3–2.3)
Muffin (*keksi*)	4	76	(58–99)	138	(38–252)	144	(59–237)	2.2	(0.4–3.9)
Popcorn	4	63	(63–63)	73	(0–122)	111	(89–138)	1.2	(0.0–2.3)
Wafers	4	54	(52–56)	111	(63–153)	134	(60–165)	1.7	(0.7–3.4)
Homemade food									
*Belyashi*	4	119	(95–137)	458	(363–539)	163	(143–195)	4.8	(3.5–5.8)
Bread (*Baursak*)	4	63	(49–74)	292	(192–395)	75	(49–96)	7.2	(3.6–12.2)
Bread (*Lepyoshka*)	4	60	(43–70)	261	(227–322)	94	(61–109)	4.9	(3.7–6.3)
Bun	4	96	(35–146)	165	(28–453)	120	(38–182)	2.7	(0.4–5.9)
Cake	5	118	(61–190)	260	(51–551)	141	(35–355)	3.4	(1.8–5.5)
*Chebureki*	4	124	(103–139)	642	(447–850)	181	(147–211)	6.3	(3.7–9.0)
Corn cob	4	220	(151–385)	158	(3–429)	500	(320–841)	0.4	(0.0–0.9)
Ice-cream	4	132	(93–166)	69	(39–121)	218	(119–308)	0.5	(0.4–0.7)
*Jent*	4	116	(60–60)	11	(5–15)	70	(40–100)	0.3	(0.1–0.5)
*Kozhe*	4	200	(200–200)	657	(591–757)	69	(43–104)	18.7	(10.2–26.1)
*Kurut*	4	31	(22–40)	1,084	(818–1,278)	78	(58–106)	25.1	(16.9–34.3)
Lagman	4	524	(438–635)	2,248	(1,577–3,735)	964	(369–1976)	6.6	(1.7–17.2)
Pancakes	4	107	(77–136)	340	(214–444)	144	(72–196)	4.2	(3.4–5.0)
*Pirozhki*	4	123	(111–130)	437	(390–498)	185	(152–222)	4.0	(3.6–4.4)
*Plov*	4	406	(352–479)	2084	(1,465–2,692)	758	(460–989)	5.0	(3.8–7.8)
Salad (cabbage)	4	90	(69–112)	601	(436–740)	156	(144–171)	6.5	(5.1–7.5)
*Samsa*	4	96	(79–112)	475	(338–842)	144	(81–179)	7.1	(3.4–17.7)
Sausage roll	4	99	(67–134)	633	(421–734)	120	(84–149)	9.0	(7.9–10.6)
*Shashlik*	4	96	(58–126)	644	(242–837)	343	(211–499)	3.2	(1.9–5.0)
*Shawarma/Doner* k*ebab*	4	385	(353–429)	1707	(1,357–1983)	1,327	(1,179–1,561)	2.2	(1.9–2.9)

Na, sodium. K, potassium. Na/K, sodium-potassium ratio.

## Discussion

Street foods were widely available in *Almaty*, Kazakhstan, especially in stationary vending sites. Homemade foods were more frequent than industrial foods, of which the most common were main dishes, sandwiches, savoury pastries and bread. The most frequent industrial foods were snacks, chocolate and confectionery, as well as sweet pastries, bun, cakes and cookies. Traditional homemade dishes and savoury pastries presented large serving sizes and were high in total fat, SFA, TFA and sodium. Industrial foods were more energy-dense than homemade foods, but no significant differences were found in the energy content per serving.

The industrial and homemade street foods analysed were found to have high (mean: 4.5 kcal/g) and medium (mean: 2.8 kcal/g) energy density, respectively. The most energy-dense items were mainly savoury and sweet snacks and cakes, industrial (chips: 5.5 kcal/g; chocolate: 5.3 kcal/g) and homemade (*jent*: 5.0 kcal/g; cakes: 4.0 kcal/g). For most of these items, over 90% of their energy was due to their fat and carbohydrates content. These food groups are commonly found in streets in LMIC worldwide; their high content in sugar and fat has been described [2, 26–28], as well as the higher energy density of ultra-processed foods [29]. An additional concern regarding the energy density of the most commonly available street foods in *Almaty* is raised when observing that only three of the 30 sampled street foods were very low or low energy-dense: homemade *kozhe,* a cereal-based traditional drink (mean: 0.2 kcal/g), cabbage salad (mean: 1.0 kcal/g) and boiled corn cob (mean: 1.1 kcal/g).

Although traditional homemade dishes presented the highest mean energy content per serving, they were also the street foods with highest protein contribution to their total energy (11–23%). These foods are essentially meat-based, as observed in other settings [27, 28, 30], and concurrent with the documented high availability and meat consumption in this region [6]. In addition, they are usually eaten at main courses and in larger servings, compared with other street foods which are expected to be consumed as snacks. Adding to similar observations in LMIC countries [2], street food may have, thus, an important role in the protein intake of this urban population. This is particularly relevant given that a high fat content was also found in the foods pertaining to these groups. Regarding the lipid profile specifically, both industrial foods, as expected [29], and also many homemade foods were high in SFA and TFA. One serving of *shashlik* (meat dish)*, jent* (sugar and oil-based sweet) and *kurut* (dairy snack) accounted, respectively, for 35%, 18% and 16% of their energy due to SFA. While in *shashlik* and *kurut*, SFA may be naturally present, in *jent* it might indicate the use of unhealthy fat ingredients rich in these fatty acids. The types and quantities of fats used to cook these foods are expected to vary between vendors [2, 3, 31], which might influence the variability found among samples within the same food. Moreover, only one third of the analysed street foods [industrial chocolate, chips and popcorn; homemade cabbage salad, *shawarma* (meat dish), pancakes, *piroshky*, *chebureki*, meat-based fried pastries*,* and *lagman,* a vegetable and meat-based dish] complied with the limit of 2 g TFA/100 g fat defined by the Eurasian Economic Commission to be applied in fat products used in the manufacture or preparation of foods [32]. Although the highest content was found in one sample of homemade cake (42 g TFA/100 g fat), it should be highlighted that one sample of *lepyoshka*, a typical flatbread in Central Asia, also presented 39 g TFA/100 g of fat. Even though this legislation was not in place when this study was conducted, these findings advocate for the need to address monitoring actions at large and small-scale production levels to attain compliance with national and international standards.

The main sources of sodium were also traditional homemade dishes. One single serving of *plov* and *lagman* overcame, respectively by 4% and 12%, the maximum daily WHO recommended intake of 2,000 mg [33]. Similar findings were described in the capital cities of two countries in Central Asia, Tajikistan and Kyrgyzstan, where traditional dishes were the leading sources of this nutrient [34]. A high content was also found in traditional snacks, such as *kurut*, or fried pastries, such as *samsa, piroshky, chebureki or belyashi*. One of the possible reasons underlying these findings might be the “Silk Road” pattern, described by the use of salt for food preservation, which strongly remains in the food culture nowadays [34]. The mean sodium intake in the *Almaty* population is more than triple the recommended (average of 17,200 mg of salt, or 6,774 mg of sodium). The same study concluded that the population was aware of the adverse health effects of high salt intake, despite only 10% knew that their intake was too high [35]. In addition, most homemade and industrial street foods collected in the current study do not conform to the recommended Na/K ratio below one [33]. Adding to the low availability of ready-to-eat fruits observed in the street food environment of Almaty, this is a concerning fact given that over 80% of the Kazakh population aged 15 years and older does not meet the daily recommendation for consumption of fruits and vegetables [36], which are important sources of potassium. The inclusion of the improvement of the population’s awareness of a healthy diet into national health policies might lead to better food choices among consumers, namely at the street food environment level.

The low contribution to nutritional quality in the studied foods also extends to the availability of ultra-processed foods [37], namely soft drinks (in over half of the street food vending sites selling beverages). These are among the dominant products in TV food advertising [35] in the country and one of the most widely available ultra-processed foods in Asia [38], which consumption is expected to follow the increasing trend observed in recent years in countries in Central Asia [39, 40]. Nevertheless, lower energy-dense traditional beverages, such as *kozhe*, of considerable protein content, were concurrently available in urban *Almaty*. These findings suggest that, in contrast with other settings where westernised products, including ultra-processed options, were widely available [2, 30], a much higher availability of traditional cooked street foods was found in cities in Central Asia, *Almaty*, *Dushanbe* and *Bishkek* [27, 28]. Therefore, although street food vendors and small manufacturers may hold a great power in the food service sector in urban Kazakhstan, it should be valued and improved to counterbalance the great influence of globalisation on the nutrition transition currently occurring in the region [6, 38].

Kazakhstan is committed to implement salt reduction and TFA elimination initiatives, product reformulation and labelling, standards for public provision of food, public awareness and improved availability of fresh fruits and vegetables [35]. The current findings reinforce the potential role of the local street food environment in such multi-pronged strategies. The organization of the street food vending sites in public markets and under supervision of local authorities, usual in urban Asia [27, 41], could enable the collaboration between them and vendors in the scope of monitoring actions regarding the sodium and TFA content of food. In addition, the implementation of education actions aiming to raise awareness about excess intake of these nutrients and healthier food practices or the provision of incentives to vendors and local producers, in order to increase the use of fresher and healthier ingredients, particularly fats might be complementary initiatives. These would lead to the improvement of the nutritional composition of street food [42], while valorising minimally processed foods and freshly prepared dishes, ultimately promoting the local gastronomy.

### Strengths and Limitations

One of the limitations of this study is the fact that it was conducted in the summer, and the observed number and diversity of street food vending sites could be higher than in other seasons, given that a substantial proportion of the informal vendors reported not working in adverse weather conditions. In addition, it has been previously reported that street foods may vary with agricultural seasonality [1]. Nevertheless, in this setting, we expect that seasonality might affect mostly the availability of some ingredients used in the preparation of homemade street foods (e.g., fillings of sweet and savoury pastries and ingredients of main dishes), but not the availability of the identified food groups, nor their nutritional composition in terms of sugar, salt and fat. Routine application of this methodology would be an important tool for monitoring various characteristics of the street food environment [43] while minimising the issue of seasonal variability. It would also allow to monitor and compare the effects of possible changes in food availability due for example to shocks in the food system, as observed during the COVID-19 pandemic [44].

Despite the limitations inherent to a cross-sectional description of Almaty’s street food environment in the summer of 2017, it is noteworthy that this study adds comprehensive insight into the characterization of street food environments in more urbanized settings [15], and in this world region, where a lack of diet-related surveys have been conducted [8, 16]. The stepwise methodology for data collection and analysis has an extensive potential of adaptability to different settings [19], allowing comparison of results, although the generalization of the findings is limited due to local cultural specificities. The estimation of the nutritional composition of the analysed street foods through reliable chemical analysis methods is another strength, which overcomes previous limitations in street food research [2, 15, 45].

### Conclusion

The wide availability and variety of street foods found in *Almaty* seem to illustrate its relevance for this urban population. Industrial foods were more energy-dense than homemade foods, but no significant differences were found regarding energy per serving. Meat-based traditional homemade dishes and pastries were predominant sources of protein and particularly high in total fat, namely SFA and TFA and sodium. The findings of this study call for the implementation of health promotion strategies targeted at vendors, consumers and several stakeholders, in order to improve the nutritional profile of street foods, ultimately addressing NCDs and associated health inequities in the urban context.
