# Supplementary material for: Availability and Nutritional Composition of Street Food in Urban Central Asia: Findings From Almaty, Kazakhstan
Source: Int J Public Health. 2022 Apr 25;67:1604558. doi: 10.3389/ijph.2022.1604558 (PMC9081345; doi:10.3389/ijph.2022.1604558)
Supplement: Supplementary file 1 [file Table1.docx]

| **Supplementary Table 1. Characteristics of street food vending sites and food offer, by type of vending site (n=384) (Almaty, Kazakhstan, 2017)** | | | | | | | |
| --- | --- | --- | --- | --- | --- | --- | --- |
|  |  | | **Type of vending site** | | | |  |
|  | **Total**  **(n=384)** | | **Stationary**  **(n=356; 92.7%)** | | **Mobile**  **(n=28; 7.3%)** | | **p** |
| **Vendors and vending sites characteristics** | **n** | **%** | **n** | **%** | **n** | **%** |  |
| **Food vendor sex (women)** | 285 | 74.2 | 265 | 74.4 | 20 | 71.4 | 0.726 |
| **Food vendor ownership** | 126 | 32.8 | 106 | 29.8 | 20 | 71.4 | <0.001^c^ |
| **Access to drinking water** | 384 | 100.0 | 356 | 100.0 | 28 | 100.0 |  |
| **Access to toilet facility** | 381 | 99.2 | 353 | 99.2 | 28 | 100.0 | 0.626 |
| **Food availability** |  |  |  |  |  |  |  |
| **Fruit** | **4** | **1.0** | **4** | **1.1** | **0** | **0.0** | **1.000** |
| **Food other than fruit** | **356** | **92.7** | **333** | **93.5** | **23** | **82.1** | **0.026 ^c^** |
| **Preparation of homemade foods ^a^** |  |  |  |  |  |  |  |
| Cooked | 185 | 79.1 | 163 | 77.3 | 22 | 95.7 | 0.039 ^c^ |
| Uncooked | 86 | 36.8 | 80 | 37.9 | 6 | 26.1 | 0.264 |
| **Beverages** | **182** | **47.4** | **166** | **46.6** | **16** | **57.1** | **0.283** |
| Water | 104 | 57.1 | 103 | 62.1 | 1 | 6.3 | <0.001 ^c^ |
| Soft drinks | 102 | 56.0 | 102 | 61.5 | 0 | 0.0 | <0.001 ^c^ |
| Traditional beverages ^b^ | 96 | 52.8 | 86 | 51.8 | 10 | 62.5 | 0.413 |
| Energy drinks | 55 | 30.2 | 55 | 33.1 | 0 | 0.0 | 0.003 ^c^ |
| Tea | 55 | 30.2 | 39 | 23.5 | 16 | 100.0 | <0.001 ^c^ |
| Coffee | 46 | 25.3 | 32 | 19.3 | 14 | 87.5 | <0.001 ^c^ |
| Fruit juice-based drinks | 39 | 21.4 | 39 | 23.5 | 0 | 0.0 | 0.025 ^c^ |
| Milk | 20 | 11.0 | 20 | 12.1 | 0 | 0.0 | 0.223 |
| Fruit smoothies | 13 | 7.1 | 13 | 7.8 | 0 | 0.0 | 0.609 |
| Fresh fruit juice-based drinks | 5 | 2.8 | 5 | 3.0 | 0 | 0.0 | 1.000 |
| ^a^ The sum of the values for this variable is higher than the total number of homemade foods, as each vendor could offer foods prepared in different ways. | | | | | | | |
| ^b^ Traditional beverages: non-alcoholic - *ayran* (n=43), *kompot* (n=24), *kozhe* (n=12),  *kephyr* (n=10) and *yoghurt* (n=10); low-alcohol content - *kymys* (n=17), *shubat* (n=17) and *kvas* (n=12).  ^c^ Statistically significant differences according to Pearson’s Chi-square test, for a confidence level of 95% (p-value<0.05). | | | | | | | |

**The urban street food environment in Central Asia: findings from Almaty, Kazakhstan**
