# Supplementary material for: Availability and Nutritional Composition of Street Food in Urban Central Asia: Findings From Almaty, Kazakhstan
Source: Int J Public Health. 2022 Apr 25;67:1604558. doi: 10.3389/ijph.2022.1604558 (PMC9081345; doi:10.3389/ijph.2022.1604558)
Supplement: Supplementary file 2 [file Table2.docx]

**Supplementary Table 2. Examples of traditional foods collected at street food vending sites (Almaty, Kazakhstan, 2017)**

| **Industrial street foods** | | |
| --- | --- | --- |
| Muffin/ *keksi* | 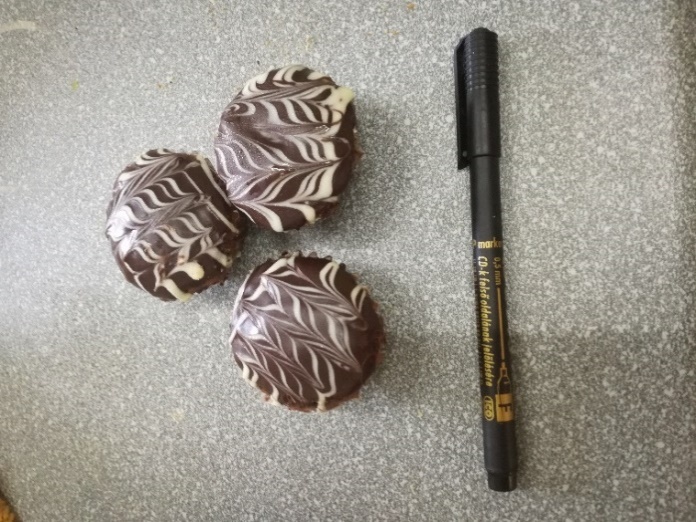 | |
| Gingerbread cookie/ p*ryaniki* | 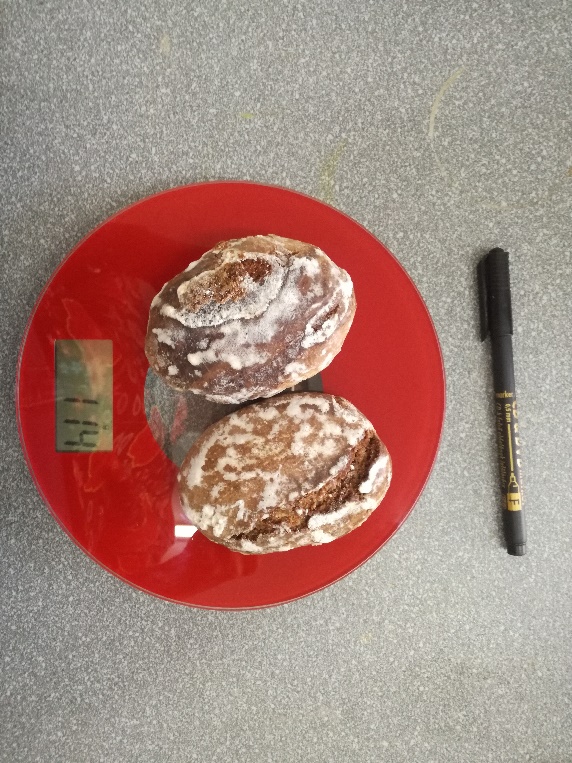 | |
| **Homemade street foods** | |  |
| *Belyashi:* small meat pie | 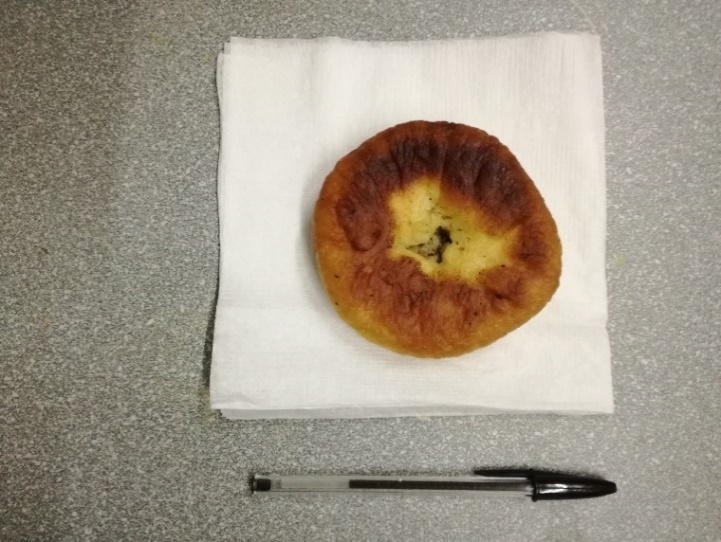 |  |
| *Bread (baursak):* fried puffy bread | 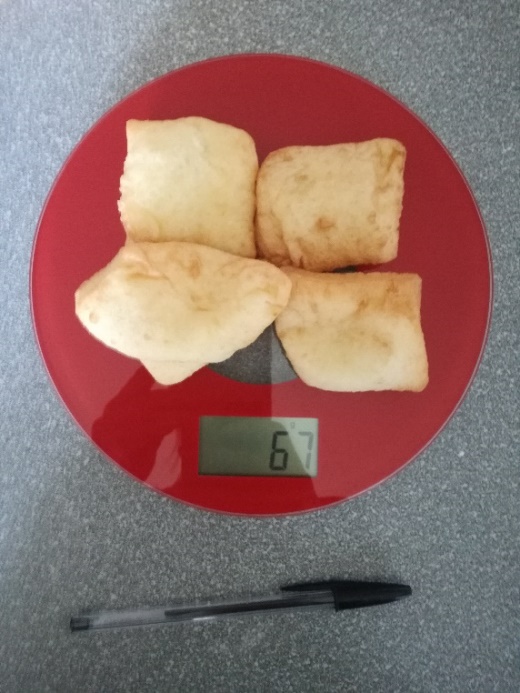 |  |
| *Bread (lepyoshka):* flatbread | 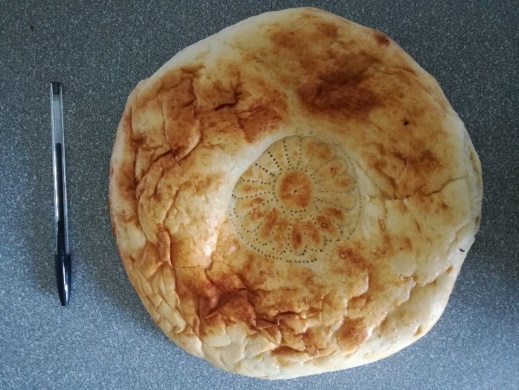 |  |
| Bun | 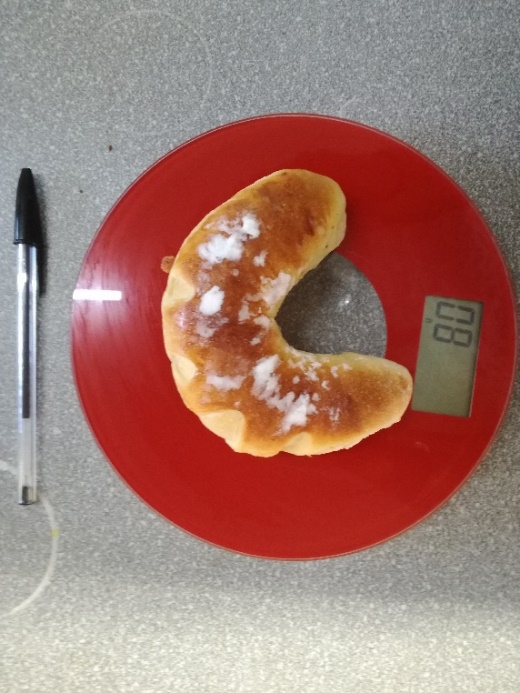 |  |
| Cake | 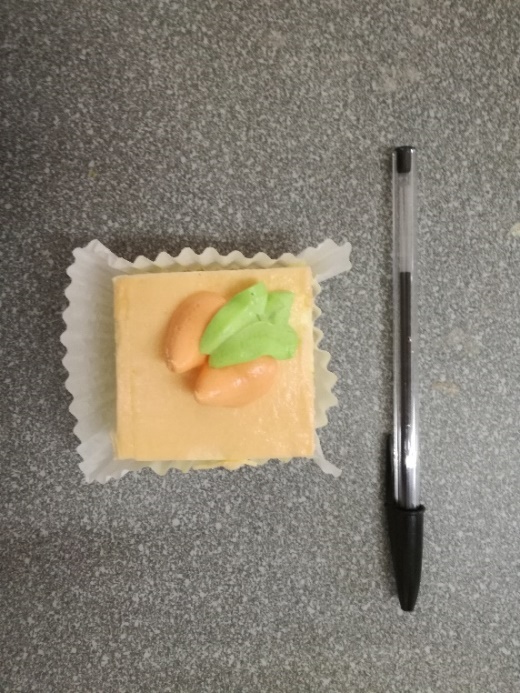 |  |
| *Chebureki*: fried savoury pastry generally filled with ground or minced meat and onions | 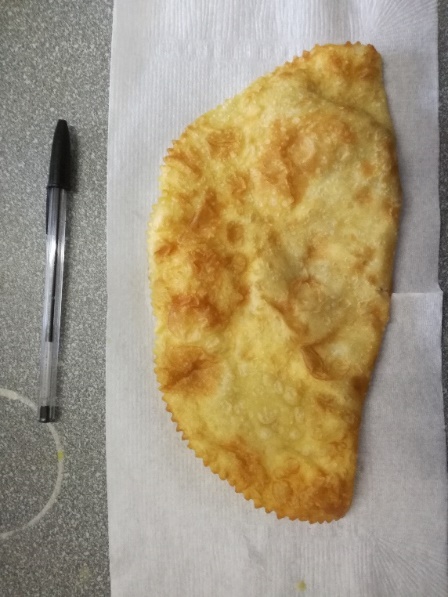 |  |
| Corn cob | 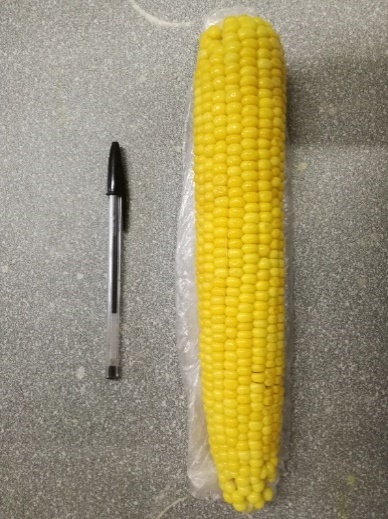 |  |
| *Jent:* dessert made of a hard grain like millet, soaked in sugar and oil | 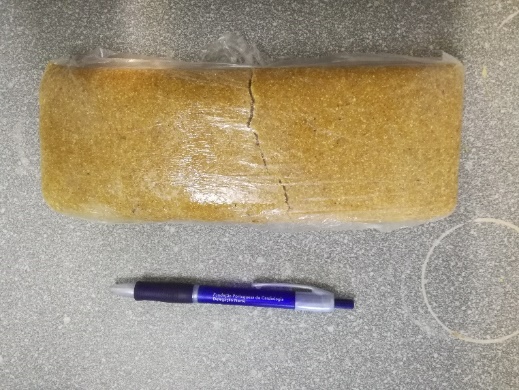 |  |
| *Kozhe:* cold drink made by boiling rice, millet or pearl barley with a mixture of dairy products such as *ayran* (fermented beverage made from sheep's milk, with salt) or *kefir* (fermented milk drink made with a yeast or bacterial fermentation starter of kefir grains). Additional ingredients include salt, water, milk or meat. | 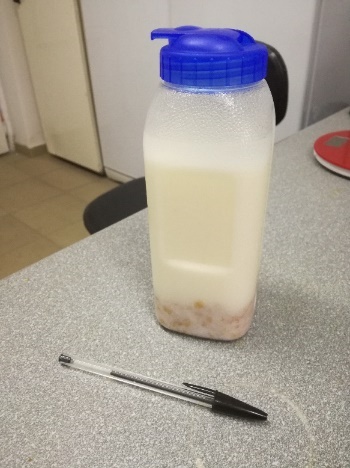 |  |
| *Kurut:* salty snack made by straining and drying sour milk or yoghurt | 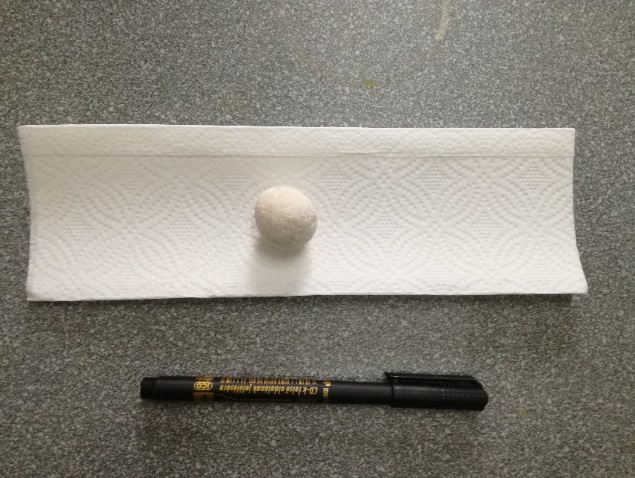 |  |
| *Lagman:* noodle dish made of chopped peppers and other vegetables, served in a spicy, vinegary sauce | 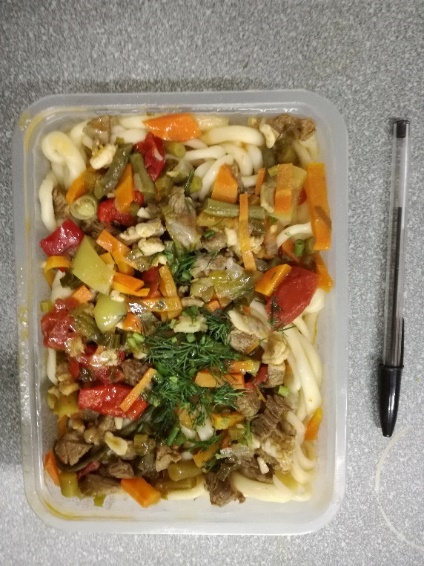 |  |
| *Pirozhki:* baked yeast pastry commonly stuffed with meat (typically beef) and/or vegetables | 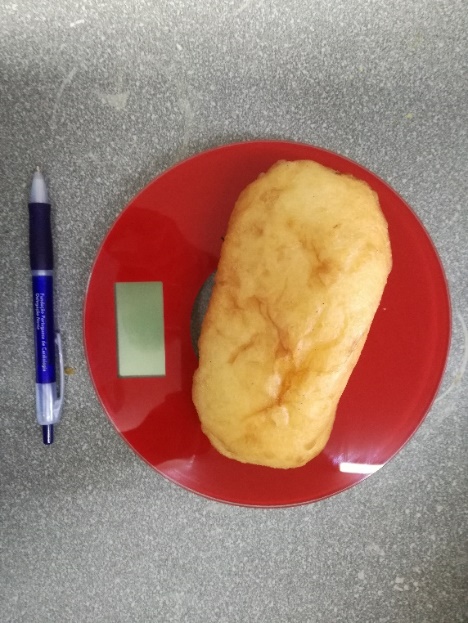 |  |
| *Plov:* rice cooked in a seasoned broth with a mixture of spices, vegetables and other ingredients, such as meat, fish and/or dried fruit | 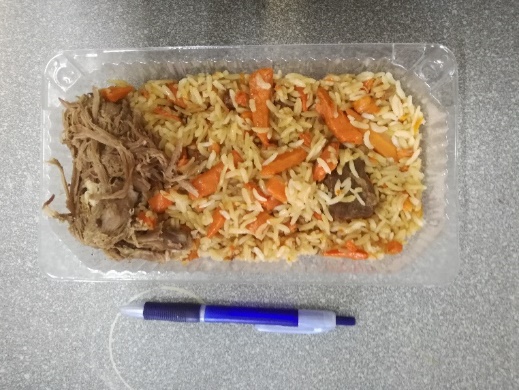 |  |
| Salad (cabbage) | 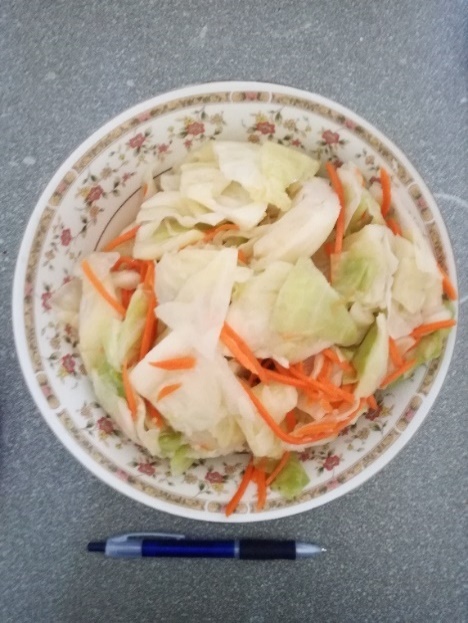 |  |
| *Samsa (also known as sambusa or samosa):* baked puff pastry usually filled with ground meat (lamb, beef or chicken) and vegetables | 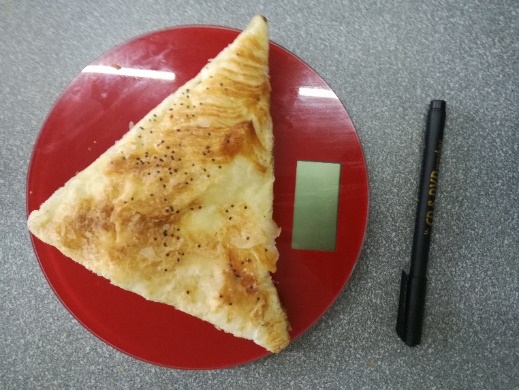 |  |
| *Shashlik:* skewered grilled cubes of meat, alone or with alternating pieces of meat, fat and vegetables (*e.g.* bell pepper, onion, mushroom, tomato) | 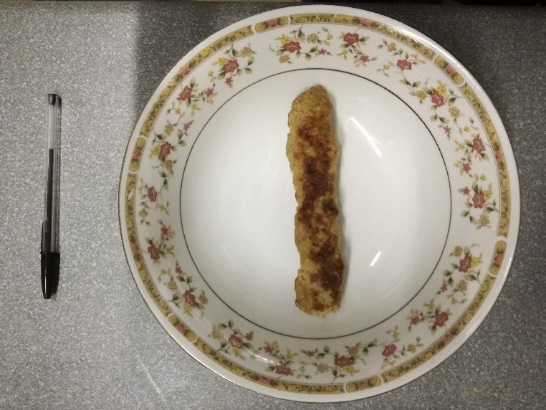 |  |
| *Shawarma/doner kebab:* seasoned meat stacked in the shape of an inverted cone turned slowly in front of a heat source | 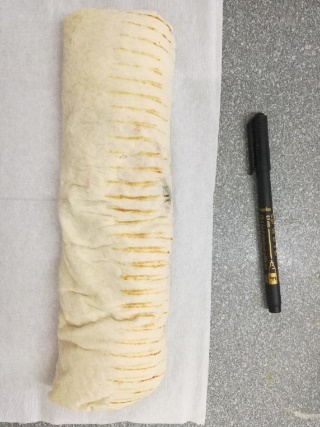 |  |
